# West Coast Boils Due to Tumbu-Fly Larvæ

**Published:** 1909-04-10

**Authors:** 


### Tropical Diseases.

### WEST COAST BOILS DUE TO TUMBU-FLY LARVÆ.

Boils and sores of various sorts are of widespread occurrence in the tropics, and their cause varies considerably with the locality. On the West Coast of Tropical Africa, particularly in Sierra Leone, there is a disease of which the lesions are so like furunculosis that they are apt to be mistaken both by patients and practitioners for ordinary boils, though they are really due to the presence in the subcutaneous tissues of the larvæ of the Tumbu fly. A good account of the disease is given by Major Blenkinsop, R.A.M.C., in the *Journal of the R.A.M.C.* (1908, p. 16).

In the majority of cases only a single larva is found in an individual at one time, though as many as fifteen and even twenty-four have occasionally been recorded. In Europeans the upper parts of the thigh and the buttock are the favourite sites for the larvæ, and it is generally held that the parasites are often acquired at the latrine. Other parts than the thigh and buttock may be affected, however, the forearm, for example, or such other exposed regions. Major Blenkinsop records one case in which the patient, a European, was in the habit of lying on his bed in an almost nude condition during the heat of the day, with the result that the characteristic sores appeared simultaneously on the chest, back, and legs. Among the partly clothed natives of Sierra Leone the site of the lesion may be very varied.

Each place takes from ten days to a fortnight to attain full development. During the earlier stages there is so little inconvenience that the patient's notice is not attracted to it, and consequently the actual history given is usually shorter than ten days. Though extremely painful sometimes, the sores do not incapacitate a man for work unless they affect the axilla or excite secondary inflammation of lymphatic vessels and lymphatic glands. When developed, a small central area apparently of suppuration is to be seen surrounded by an areola of inflammation from three-quarters of an inch to an inch in diameter. On careful examination of the central area some black matter, the excrement of the larva, may be seen immediately beneath the skin, and in the centre a minute breathing aperture. If the latter is very gently pressed with the tip of the finger intense pain is caused, very possibly on account of movements of the larva. In some cases in which secondary pyococcal infection occurs an abscess is produced.

In ordinary cases the pain, though severe, is paroxysmal, and the intense continuous throbbing of the large furuncles is absent. If allowed to run its course, the larva leaves its nidus spontaneously when fully developed. It does not burrow deeper than the subcutaneous tissues.

The following are the points upon which Major Blenkinsop lays particular stress in differentiating the sores of Tumbu fly disease from boils: (1) The presence of the black excrement around the larva's breathing aperture; (2) the pain caused by gentle and continuous pressure on the breathing aperture; (3) the paroxysmal character of the pain, which is generally unaccompanied by throbbing.

The larva can usually be removed entire with the point of a surgical needle. If uninjured, it is very active for some time after its removal. The small wound heals rapidly if washed out with some antiseptic lotion, such as carbolic acid 1 in 20 and then covered with an antiseptic dressing. A small depressed and more or less pigmented scar results. A free incision may be required to let out pus.

In one case under Major Blenkinsop's care, in which there were several larvæ present at the same time, the patient had applied a plaster of sugar and soap to some of the sores, and the larvæ appeared on the surface in less than twelve hours in every case he had thus treated.

